# Beyond Obesity and Lifestyle: A Review of 21st Century Chronic Disease Determinants

**DOI:** 10.1155/2014/731685

**Published:** 2014-04-07

**Authors:** Garry Egger, John Dixon

**Affiliations:** ^1^School of Health and Human Sciences, Southern Cross University, P.O. Box 313, Balgowlah, Lismore, NSW 2093, Australia; ^2^Clinical Obesity Research, Baker IDI Heart and Diabetes Institute, Melbourne, VIC, Australia

## Abstract

The obesity epidemic and associated chronic diseases are often attributed to modern lifestyles. The term “lifestyle” however, ignores broader social, economic, and environmental determinants while inadvertently “blaming the victim.” Seen more eclectically, lifestyle encompasses distal, medial, and proximal determinants. Hence any analysis of causality should include all these levels. The term “anthropogens,” or “…*man-made environments, their by-products and/or lifestyles encouraged by these, some of which may be detrimental to human health*” provides a monocausal focus for chronic diseases similar to that which the germ theory afforded infectious diseases. Anthropogens have in common an ability to induce a form of chronic, low-level systemic inflammation (“metaflammation”). A review of anthropogens, based on inducers with a metaflammatory association, is conducted here, together with the evidence for each in connection with a number of chronic diseases. This suggests a broader view of lifestyle and a focus on determinants, rather than obesity and lifestyle *per se* as the specific causes of modern chronic disease. Under such an analysis, obesity is seen more as “a canary in a mineshaft” signaling problems in the broader environment, suggesting that population obesity management should be focused more upstream if chronic diseases are to be better managed.

## 1. Introduction

Modern western lifestyles are often blamed for the current obesity and associated chronic disease pandemics [1]. This seems plausible as such problems, at a population level, only really began 3-4 decades ago [2]. They are also not usually caused by any microbial agent and have occurred too quickly for genome changes to be a factor [2] (although this does not preclude environmental influences on gene expression). The increased aging of the population is a consideration, but increased risk factors across all age groups limit aging as a sole explanation [3]. Other behaviors [4] and environmental factors [5] have been implicated, but a single causal underpinning is illusive, thus making “lifestyle” an attractive proposition.

However, lifestyle (“a mode of life chosen by a person or group”-Macquarie Dictionary) infers volitional behavior on the part of an individual. This has a pejorative meaning for some social scientists as it is thought to ignore the deeper social, economic, and environmental determinants of both lifestyle and disease, while focusing on individual responsibility, that is, “victim blaming” [6]. A more holistic view would involve not only looking at the “cause” of a disease, but also, as Rose has pointed out in [7], looking at the “cause of the cause,” and even the “cause of the cause of the cause.” This is particularly relevant for an understanding of the chronic, noncommunicable diseases (NCDs) often linked to obesity, in contrast to infectious/communicable diseases that have prevailed historically [8].

Infectious diseases benefited from a monocausal focus [9] provided by the “germ theory,” which culminated in improvements in public health, hygiene, immunization, and the development of antibiotics in the early 20th century [10].

Chronic diseases began to replace the decline in infections in the west in the late 20th century, taking health experts somewhat by surprise. Epidemiologists noted a phase in the development of a country called the “epidemiological transition” [11] when chronic diseases take over from infections as the main disease burden. This occurred in the 1970s and 1980s for many developed economies in North America, Europe, and the Asian-Pacific region and is currently occurring in others, such as Brazil, Russia, India, and China. The double-edged sword of progress, which facilitated control over infectious diseases, instigated an unhealthy trend in chronic diseases. The worldwide rise in obesity has been an accompaniment to this.

## 2. Causality in Disease

**Figure 1:**
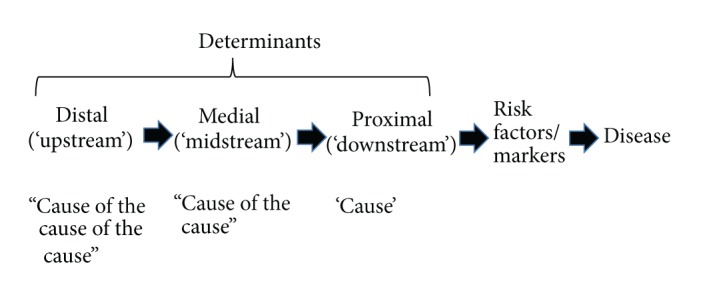
A hierarchy of determinants and risk factors/markers in chronic disease aetiology.

While infectious disease can usually be ascribed to microbial causes, causality in NCDs is more problematic. In most cases the agent of causality is ill-defined, but there are layers of influence. As shown in Figure 1, most immediate to the disease are risk factors, which, in turn, are influenced by drivers or determinants at different levels from proximal or “downstream” (i.e., more immediate to the disease) to medial and then distal or “upstream.” Heart disease for example has proximal determinants of smoking, nutrition, and inactivity, medial determinants of peer pressure and food and exercise environments, and distal determinants of economic and social policies.

Unlike the germ theory for communicable diseases, there has been no equivalent underlying link between the determinants shown in Figure 1 and chronic diseases. Such a revelation is unlikely to solve the chronic disease problem. However it could help rationalize treatment resources and mitigate the inevitable disabilities and morbidities associated with aging.

The discovery, in the 1990s, of a “new” form of inflammation, which appears to be present in many if not all chronic diseases, offers the prospect of an underlying generic disease marker pointing to determinants beyond the diverse explanations of aging and lifestyle. The term “metaflammation” [12] has been used to define a form of low-grade, chronic, and systemic inflammation, originally ascribed to obesity [13]. Subsequent research has shown that metaflammation is not limited to obesity, but associated with other lifestyle and environmental “inducers” [14], examples of which have been linked, either directly or indirectly to certain chronic diseases and conditions like heart disease, type 2 diabetes, respiratory problems, many forms of cancer, and even depression and dementia [12, 15]. Metaflammation appears to be part of a metabolic cascade, including cellular oxidative stress and insulin resistance, which induces allostatic overload, dysmetabolism, and ultimately chronic disease [16]. This raises the question of whether obesity is a* necessary* condition for the chronic diseases with which it is associated or just a* sufficient *condition in some circumstance and whether lifestyle and environmental inducers which may (or may not) lead to obesity can be independent determinants of disease as indicated by metaflammatory processes.

Inducers of metaflammation, which have largely arisen since the industrial revolution [17, 18], have been labeled “anthropogens,” or “…*man-made environments, their by-products and/or lifestyles encouraged by these, some of which may be detrimental to human health*” [19]. Anthropogens incite a low level, but persistent immune response to a not-immediately life-threatening situation, which can become dysmetabolic when exposure is chronic. Because such a response is undifferentiated, the outcome is systemic rather than localized.

An individual's susceptibility to a range of anthropogens clearly varies with genetic predisposition to chronic diseases carried through the genome including obesity, diabetes, many cancers, and cardiovascular disease. However, during the second half of the 20th century, a revolution in our understanding of environmental influences and lifelong gene expression has emphasized the importance of early environmental influences on the later development of chronic disease. It is now clear that epigenetics, the heritable changes in gene activity not driven by change in the DNA sequence, has a major influence on the susceptibility to chronic disease for both individuals and their offspring. Chronic disease driven by a mismatch between the environment one is programmed for and the environment one is born into is highlighted by the extreme susceptibility of indigenous and developing communities to anthropogens and related chronic diseases.

Obviously, not all anthropogens are unhealthy for all people. The identification of those that have negative health effects however is important for developing a focus on chronic disease aetiology. We consider that a review of anthropogens could focus the attention of health workers and hence provide such a review below. We provide evidence of metaflammatory reactions from either inadequate or excessive exposure to these and evidence for their association with a number of chronic diseases. A list of disease categories is shown in Table 1.

A summary of anthropogens associated with these disease categories, discussed in the text, is given in Table 2 using the acronym NASTIE ODOURS.

## 3. Identifying “Anthropogens”

In discussions of modern chronic disease etiology, smoking, poor nutrition, excess weight, and alcohol use stand out as the dominant preventable determinants [20]. However recent research has expanded this considerably to take account of social, cultural, occupational, environmental, and other factors (“anthropogens”) in the hierarchical structure (Figure 1). A list of these is described below. The discussion is not meant to be an extensive review of each topic area but rather to cover the main components of each identified here as being associated with chronic disease and with a common metaflammatory base. Each is also considered in its own right an independent determinant in the absence of obesity or weight gain. 

*Nutrition*. The importance of nutrition for the prevention and management of chronic disease is well known [21]. Inadequate or overnutrition has been proposed to account for up to two-thirds of risk for certain chronic problems like type 2 diabetes and cardiovascular disease [22] and a significant proportion of other chronic ailments [23]. Health problems have been related to both specific nutrients [24] and overall meal patterns [25], with inflammatory biomarkers generally accompanying those foods/eating patterns associated with disease risk in the presence and the absence of obesity [17, 26].

Excessive energy intake, particularly of high energy-dense, but low nutrient-dense products, is a major problem of industrialised societies. Still, excessive intake of even healthy foods can increase postprandial (and potentially chronic) metaflammation [27], suggesting negative long-term outcomes. At the other extreme, chronic energy restriction is now well documented as being associated with increased longevity and improved heath [28].

In relation to nutrition quality, studies have reported increased risk and elevated metaflammation from excessive amounts of sugars, salt, alcohol, and (saturated and trans) fats, as well as inadequate levels of fibre, fruit, vegetables, grains, and certain nutrients [17, 26]. Levels of processing have been proposed as a general indication of risk [29], and there appears to be a clear postprandial “metaflammatory” trail from processed versus whole foods, suggesting an evolutionary role in nutritional health [18, 30, 31]. Although individual and genetic factors influence outcomes [32], the worst-case scenario for obesity and chronic disease based on current evidence would be an excessive amount of a modern, western diet made up of highly processed foods [25]. While there may be controversy over an ideal diet (mediterranean, anti-inflammatory, paleo, etc.), Michael Pollan's [33] dictum to “Eat food. Mostly plants. Not too much”, provides a simple, concise, and accurate long-term nutritional goal. 

*(In)Activity*. Inactivity, as well as sedentary activities like sitting, in contrast to insufficient physical activity, is an independent risk factor for disease [34]. It is one of the major unhealthy anthropogens of our times with links to over 35 different diseases [35].

Movement, physical activity, and exercise can be conceived of as gradations along a scale and all have a role, to different degrees, in primary prevention of a range of diseases and, in some cases, treatment and reversal of risks and/or disease entities (namely, type 2 diabetes). This is mainly through the modems of aerobic capacity and/or muscle strength and integrity. Flexibility and balance provide musculoskeletal integrity that can enhance quality of life.

While controversies exist about type, intensity, frequency, and duration of physical activity, there is no dispute about the health value of an optimal physical activity requirement for humans. A generic prescription based on “volume” (intensity × frequency × duration) incorporating both aerobic and resistance training is appropriate in the absence of a more detailed individual-genetic understanding [36]. In the absence of this, recommendations that “…*any activity is better than none, and more is better than a little*”, and for individuals to “*think of movement as an opportunity, not an inconvenience*” [37] are appropriate. The relationship between activity and health has been referred to as a U-shaped function, with excessive exercise having diminishing health benefits as reflected in increased metaflammation, similar to that of inactivity [38, 39].

Poor nutrition and inactivity are the best-known inducers of weight gain. Several studies however now show that either poor nutrition or inactivity can independently modify metaflammation without significant changes in weight [40, 41]. 

*Stress, Anxiety, and Depression*. The nature of stress has changed in recent times from an acute warning signal to a chronic strain on physiological adaptation. Typically, the body's reaction to a stressor has been “flight” or “fight,” but these options are less viable in the modern environment, leading to chronic effects such as elevated adrenocortical hormone concentrations, activation of the sympathetic nervous system [42], ailments like heart disease [43], and accompanying vascular, metabolic, and inflammatory processes [43, 44]. Of itself, stress is not a health issue, and a certain amount within the coping capacity of the individual [45] is vital for a healthy life. It is the “strain,” resulting from excessive stress, outside the limitations of the stressee to cope, and resulting in anxiety and depression that can lead to allostasis and chronic disease.

Anxiety is a form of “feared helplessness” defined as “…*a thin stream of fear trickling through the mind. If encouraged, it cuts a channel into which all other thoughts are drained*” [46]. Anxiety occurs while an individual is striving to adapt and the association of this with ill-health is diffuse. However it is when striving ceases that depression or “learned helplessness” [47] can result, with more defined channels into a range of chronic diseases. High levels of depression have been shown to be related to a range of chronic diseases from type 2 diabetes [48] to Alzheimer's [49]. A consistent finding is a link between stress, anxiety, and depression and increased inflammatory markers, which can be associated with [50] or independent of body weight [51]. 

*Technology-Pathology*. The association of chronic disease with certain modern forms of technology is often overlooked or disregarded. This can range from death or chronic pain initiated from motor vehicle or machine injuries to auditory problems from amplified music [52]. At the extremes, it can range from mortality and morbidity from firearms and high tech weapons used in warfare to apparently obscure problems like dermatoses [53], other skin disorders [54], impaired vision [55], and repetitive strain injury from excessive computer and small screen use [56].

Other recent problems within this category are acute and chronic problems that occur while focusing on use of social media (e.g., texting and tweeting) whilst carrying out other activities, such as driving [57]. Because of its immediacy, social media bullying and intimidation can also lead to psychological morbidities and even suicide amongst prone youth, although this is not as yet well documented in the medical literature. Other problems such as “facebook depression” [58] are only beginning to emerge. Social contagion [59] effects on disease are amplified through the use of social media as shown in the association between social networks and chronic disease risks like obesity [60] and smoking [61]. Control of technology misuse is traditionally through legislative restrictions (i.e., use of cell phones while driving) but personal controls on behavior are also likely to be necessary. 

*Inadequate Sleep*. Healthy sleep is the anchor for a healthy life, thus interacting with other chronic disease determinants discussed here [62]. Together with inactivity, inadequate sleep is one of the most underrated lifestyle risk factors for chronic disease [63]. Poor sleep is associated with an increase in inflammatory markers [64], as well as more classic risk factors [65] and significant social impacts [63]. The cumulative long-term effects of sleep deprivation and sleep disorders have been associated with a wide range of deleterious health consequences including an increased risk of hypertension, diabetes, obesity, depression, heart attack, and stroke [66]. As many as 80% of people in western countries will suffer from a sleep problem at some stage in their life, 30–50% will have difficulty in sleeping [67]. According to the US National Sleep Foundation, the average of eight to nine hours sleep per night in previous years has now dropped to around seven hours per night, with 37% of young adults getting <7 hours in 2002 compared to less than half that (16%) in 1960 [68].

Modern lifestyles are often in direct competition with sleep so much so that it could be argued that the majority of modern sleep problems have a basis in lifestyle choices. The combination of sufficient sleep with other lifestyle factors (e.g., physical activity, a healthy diet, moderate alcohol consumption, and nonsmoking) has additional value in heart disease prevention than sleep alone [69]. Sleep deprivation can also indirectly affect other disease determinants. Appetitive food mechanisms in the brain for example stimulate a greater desire for “junk” food after sleep deprivation, thus potentially enhancing obesity [70]. Unfortunately chronic disease often interferes with sleep quality and quantity generating a bidirectional vicious cycle, a situation commonly encountered in chronic disease. Inadequate sleep also has a strong relationship with elevated inflammatory markers [71]. On the positive side, sleep can be dramatically improved with a healthy approach to the lifestyle and a structured approach to sleep hygiene [72]. Simple actions like the removal of interactive media from adolescent bedrooms can be a starting point for better sleep [73]. 

*Environment*. Aspects of the environment have always been a consideration in public health. However the rise of chronic diseases has led to a more structured approach to this. Swinburn et al. [74], for example, consider four types (physical, economic, policy, and sociocultural) and two sizes (micro and macro) of “obesogenic” [75] environments, which serve to draw attention away from purely biological explanations of obesity and by extension chronic disease.

Small particle pollution from exhaust and industrial fumes [76] as well as a wide range of chemicals in the air, water, soil, and households [77] makes up the natural physical environment. A large group of such pollutants, labeled endocrine disrupting chemicals (EDCs) [78], has been attributed to significant physiological and even behavioural changes such as increased hunger, which can lead to obesity [79]. Exposure data (e.g., to bisphenol A) suggests a link between this and obesity in children [80], leading to the suggestion of some chemicals being “obesogens” [81]. Increases in carbon in the atmosphere are an example of a dramatic macroenvironmental change with potential health (as well as climate change) impacts [82]. Many environmental factors have also been shown to lead to increased metaflammation as an intermediatory process with links to chronic disease [78, 83].

Sociocultural influences are reflected, for example, in attitudes to feasting in some cultures which may have been suitable in historical times but are contraindicated with the imposition of a western culture and diets. Political environments make the “rules” that allow, for example, smoking or drinking in the family or unrestricted sales of unhealthy foods and products (e.g., cigarettes) in society. Overarching all of this is the macroeconomic system, including the modern economic growth model which demands consumption that is not necessarily conducive to health [84, 85].

Recent findings relating to the gut microbiome suggest that the inner environment (“in”-vironment in contrast to “en”-vironment) should also be considered within this category [86]. Changes in the gut microbiome not only appear to result from unhealthy activities but also influence outcomes, such as obesity through better energy harvesting through a “leaky gut” [87, 88].

Some protection against unhealthy environments may be provided by positive lifestyle changes [77, 89]. It should be obvious however that significant macroenvironmental reforms through legislative change, some of which may crossover with those required to moderate climate change [90] and other environmental degradation, are necessary. 

*Occupation*. Meaningful work is an important component of good health. Generally however it is the direct effects on health and safety—exposure to machinery, chemicals, injury, and so forth—or the adverse health effects of work hours and shift work [91] and their effects on inflammation [92] that are considered. Recent concern has turned more to social factors. Job insecurity and job strain, for example, have been shown to increase the risk of heart disease (although the effect may be modest and largely explainable by socioeconomic factors [93]). Poor job satisfaction is linked to “burn out,” low self-esteem, depression, and anxiety [94] and excessive work hours to a risk of ill-health and damage to social relationships [95]. In work with the British Civil Service, Marmot and colleagues have reported on the health effects of perceived social justice [96], “burn out” [97], and social standing [98, 99] relating to occupational status. Changes in the nature and security of work in the modern world mean that both the physical and psychological components of occupations need to be considered part of a lifestyle/environmental perspective on health. Hence some forms of occupation can be seen as modern-day, chronic disease promoting anthropogens. 

*Drugs, Cigarettes, and (Excessive) Alcohol*. Drugs, both licit and illicit, are responsible for a significant and increasing degree of morbidity and mortality in modern societies. The stand-out amongst licit products is cigarette smoking and its links with cancers, heart disease, and respiratory problems [100]. Legal medications form another category of drug related mortality and morbidity. Shapiro et al. [101] categorise problematic drug use into hazardous use, substance abuse, or substance dependence. Unfortunately some of the most effective medications for disorders such as schizophrenia, depression, and certain forms of epilepsy increase hunger, weight gain, and cardio-metabolic risk [102]. Illicit drug use (and the accompanying health effects) appears to increase with increased urbanization, economic prosperity, and inequality.

Its more ambiguous outcomes make alcohol a more diverse problem. Some health and social benefits of moderate consumption [103] are difficult to weigh up against the severe health and social disruption of excessive consumption, binge drinking, social and economic costs [104], and other chronic disease outcomes [100]. Overuse of alcohol is also known to have deleterious effects on several forms of disease including cancers, although this literature is not expanded on here. While excessive alcohol intake is inflammatory, moderate intake has an anti-inflammatory effect [105]. 

*Over- and Underexposure*. While many lifestyle-related behaviours have a linear association with health (e.g., smoking and sleep), others have a “U” or “tick-shaped” relationship (e.g., physical activity, alcohol, and sleep). Exposure to ultraviolet radiation (UVR) from sunlight is a case in point. UVR is classified as a carcinogen and a major determinant for several forms of skin disorders. The incidence of melanoma, the most deadly form of skin cancers, has doubled in recent years [106, 107], although this is less common than other forms of skin cancers and photoaging [108]. Intermittent extreme exposures and sunburn, as well as chronic overexposure can have differing degrees of risk [109]. Overexposure to heat and dryness (low humidity) is also thought to have adverse effects on the skin [110]. Passive smoking is yet another form of overexposure with increased risks of chronic diseases like type 2 diabetes [111].

At the other extremes, underexposure to sunlight can lead to deficiencies in vitamin D, thus increasing risks of heart disease [112], type 2 diabetes [113], and depression [114], as well as more well-known problems such as rickets [115]. Underexposure to daylight can also have unhealthy consequences in seasonal affective disorders (SAD) suffered at extreme latitudes [116]. 

*Relationships*. The quality of personal and social relationships is clearly linked to chronic disease outcomes [117] including heart disease [118], stroke [119], some cancers [120], and all-cause mortality [121].

The pathways for this are, as yet, unclear and psychological mediators have not been proven [122], but inflammatory processes have been associated with poor social relations [123] such as spousal ambivalence [124] and isolation [125] and can even stem back to maternal separation in childhood [126]. People who have supportive close relationships have lower levels of systemic inflammation compared to people who have unsatisfactory relationships [127]. Negative and competitive social interactions can even increase proinflammatory cytokine activity on a daily level [128]. In reverse, a Finnish study has shown that social support can alleviate the inflammation associated with childhood adversities [129]. Improving awareness of the importance of social support and assisting in finding such support should be integral to chronic disease management. 

*Social Disadvantage*. Social disadvantage is associated with diseases like type 2 diabetes [130] and cardiovascular disease [131]. Disadvantage exists not only through socioeconomic status [98] and income inequalities [132] but also through economic stress and security, with metaflammation as a possible link [133].

According to the Commission on Social Determinants of Health [134], inequities in power, money, and resources are responsible for much of the inequalities in health within and between countries. While the effects of socioeconomic status on health (and inflammation) are relatively clear [135, 136], the effects of income disparities on health have been more controversial. Wilkinson and Pickett [132] in a descriptive analysis of ratios of rich to poor within and between OECD countries show a linear worsening of a number of health and social problems (obesity, infant mortality, teenage pregnancies, etc.) in countries with greater income gradients.

**Figure 2:**
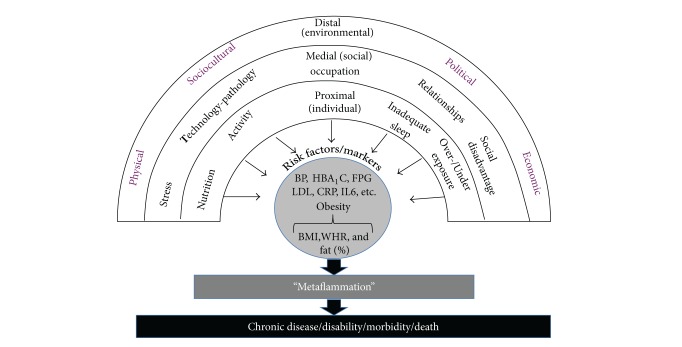
The link between “anthropogens” obesity, metaflammation, and chronic disease. While obesity is often a correlate, this does not always imply causality in chronic disease aetiology.

Much has been made of the mechanisms underlying social disadvantage, socioeconomic status, and inequality. Stringhini et al. [137] show that modifiable health behaviours and obesity could explain around 50% of the incidence in type 2 diabetes. Increases in inflammatory processes are also common with social disadvantage in different forms [138, 139] (see Figure 2).

## 4. Discussion

We have categorized a number of determinants of modern chronic diseases within a hierarchy using the acronym NASTIE ODOURS (Table 2). In doing so, we have extended the concept of lifestyle to include broader aspects of the social, political, and economic environments. Because of their man-made nature (in contrast to the microbial nature of infectious disease determinants), these have been collectively called anthropogens [19]. In addition, we have shown that most of the anthropogens discussed here have a common physiological link through chronic, systemic inflammatory (metaflammation) processes.

An obvious omission from our classification is obesity. There are two reasons for this. In the first place, obesity should be seen as a risk factor, rather than a primary determinant of disease, which is downstream, sometimes, but not always resulting from some of the determinants considered here, like overnutrition, inactivity, stress, social pressure, and so forth. A second reason is the variability of causal links between obesity and disease as exemplified in the “obesity paradox” [140] and metaflammatory associations with disease preceding or in the absence and presence of obesity [15, 17, 18]. Hence obesity is probably more a “canary in a mineshaft,” warning of problems in the broader environment than a universal cause of disease [141, 142].

There are a number of implications stemming from this discussion. Firstly, arguments about the best “diets” for weight loss and chronic disease become less relevant when looking at this big picture pattern of disease [84]. Secondly, the presence of independent upstream determinants means that weight loss should not be the sole focus of chronic disease management. Losses can be expected from changing aspects of the NASTIE ODOURS formula, but weight loss should not be seen as the sole driver of the process. Third, it would be wrong to assume that chronic disease determinants should be managed singly. The interactive nature of these determinants suggests more of a “systems” model approach to managing chronic disease problems than is often considered. Inadequate sleep and resultant fatigue, for example, can lead to a reduction in physical activity and a change in dietary patterns, uptake of technology-based entertainment, increased obesity, and resultant depression, which can continue and/or widen the cycle. The use of a “diet” for treating obesity, when underlying inflammatory processes may be related to all these more obscure determinants, is unlikely to provide optimal health. Finally a concentration on lifestyle through simply proximal and even medial determinants as defined here is unlikely to significantly influence the problem while more dominant upstream determinants remain. Effective management of modern chronic disease thus needs to be broadened to encompass a greater sphere of influence than is often publically perceived or politically popular.

## Figures and Tables

**Table 1:** Chronic disease categories with lifestyle/environmental determinants.

(1) Cardio- and cerebrovascular diseases	
(2) Cancers with lifestyle component	
(3) Endocrine/metabolic disorders	
(4) Gastrointestinal diseases	
(5) Kidney disease	
(6) Mental/CNS health	
(7) Musculoskeletal disorders	
(8) Respiratory diseases	
(9) Reproductive disorders	
(10) Dermatological disorders	

**Table 2:** Lifestyle and environmental determinants (“anthropogens”) for chronic disease. Numbers refer to chronic disease categories (from Table 1) for which there is supporting evidence referred to in the text.

Determinants	Decreases risk	Increases risk	Moderators
Nutrition 1, 2, 3, 4, 5, 6	Fruit/vegetables Dietary fibre Whole grains Food variety Seafood Healthy eating patterns	High total energy High energy density Excess processed foods High GI foods Sat./trans fats Sugars Salt Excessive alcohol Sugared soft drinks Processed/red meat	Binge eating/drinking Social/holiday eating “Restrained” eating Feasting Culture Habits

(In)Activity 1, 2, 3, 6, 7, 8, 9	Aerobic exercise Resistance exercise Stretching Stability Leisure activity Incidental activity	Sitting/sedentary work Overexercise	Fear of crime Fatigue/laziness Discomfort/injury/ Early experiences Energy-saving devices Obesity Habits

Stress, anxiety, and depression 1, 3, 4, 9	Exercise/fitness Healthy nutrition Perceived control Self-efficacy Coping skills Meaning	Overload “Learned helplessness” Early trauma Boredom Caffeine/drug use	Peer/social/pressure Uncontrollable thoughts Worry Fear of the unknown Obesity

Technology-induced-pathology 7, 10		Motor vehicle use Machinery TV/small screens Repetitive actions Noise pollution Processed foods Weapons of war	Peer/social pressure Legislation/regulation Habits

Inadequate sleep 1, 3, 6, 10	REM sleep Bed-time Hypersomnia Nutrition Exercise/fitness	Stress Entertainment Sleep disorders Overheating Interactive media Alcohol/drugs	Activity before sleep Stress Anxiety/depression Obesity habits

Environment 2, 3, 6, 9, 10	Political/economic structure Recreational space “Green” exposure Infrastructure for walking and cycling Plant-based nutrition	Passive influences Second-hand smoke Particle pollution Endocrine disrupting Chemicals (EDCs) Home chemicals Drug-immunity (e.g., antibiotics)	Social proof “Tipping point” Social/peer pressure Cultural influences Habit

Occupation 1, 2, 8, 10	Social justice Work equality Security of employment	Work stress Shift-work Hazard exposure Conflict	Peer pressure Bullying

Drugs, smoking, and alcohol 1–10	Appropriate medication	Recreational drugs Cigarette smoking Alcohol use Iatrogenesis	Stress, anxiety, and depression Peer/social pressure Addiction Binge drinking Habit

Over- and underexposure 1, 2, 3	Sunlight light stimulation	Sunlight (excess) Sunlight (inadequate) Low humidity/ asbestos Radiation	Peer/social pressure Cultural influences Habit

Relationships 1, 3, 6	Companionship Peer support Maternal support in childhood “Love”	Interpersonal conflict Loneliness Lack of support	Peer pressure Early experience

Social factors 1–10	Trust Income security Market regulation SE status Education	Inequality Poverty Deregulated markets	Stress Bullying Cognitive processes Peer/social pressure
